# Utility of Ki67 labeling index, cyclin D1 expression, and ER-activity level in postmenopausal ER-positive and HER2-negative breast cancer with neoadjuvant chemo-endocrine therapy

**DOI:** 10.1371/journal.pone.0217279

**Published:** 2019-05-21

**Authors:** Sasagu Kurozumi, Yuri Yamaguchi, Hiroshi Matsumoto, Masafumi Kurosumi, Shin-ichi Hayashi, Takaaki Fujii, Jun Horiguchi, Ken Shirabe, Kenichi Inoue

**Affiliations:** 1 Department of General Surgical Science, Gunma University Graduate School of Medicine, Gunma, Japan; 2 Division of Breast Surgery, Saitama Cancer Center, Saitama, Japan; 3 Research Institute for Clinical Oncology, Saitama Cancer Center, Saitama, Japan; 4 Department of Pathology, Saitama Cancer Center, Saitama, Japan; 5 Department of Molecular and Functional Dynamics, Tohoku University, Miyagi, Japan; 6 Department of Breast Surgery, International University of Health and Welfare, Chiba, Japan; 7 Division of Breast Oncology, Saitama Cancer Center, Saitama, Japan; University of South Alabama Mitchell Cancer Institute, UNITED STATES

## Abstract

In this study, we investigated the relationships of pathological response after neoadjuvant chemo-endocrine therapy with alterations in the Ki67 labeling index (LI), expression of cyclin D1 (CCND1) and progesterone receptor (PgR), and estrogen receptor (ER) activity in breast cancer. A total of 43 Japanese post-menopausal ER-positive and human epidermal growth factor receptor 2-negative invasive breast cancer patients with tumors >2 cm or positive lymph nodes were enrolled. Exemestane alone was administered for 2 months. Neoadjuvant chemo-endocrine therapy included four cycles of doxorubicin plus paclitaxel followed by weekly paclitaxel. The immunohistochemical expression of Ki67 LI, CCND1, and PgR, and ER activity were evaluated using core needle biopsy samples at the pretreatment and post-exemestane alone stages. ER activity was evaluated through transfection of an adenovirus vector carrying an estrogen-response element-green fluorescent protein gene. In current study, marked pathological responses (including 4.7% with pathological complete response) were obtained in 34.9% of patients. Ki67 LI and PgR expression were significantly decreased after treatment. High Ki67 LI at pretreatment was a significant predictive factor of marked pathological response. At the stage of post-exemestane alone, Ki67 LI was not significantly associated with pathological response; however, high CCND1 expression was significantly correlated with high Ki67 LI. Moreover, low-level ER activity at the post-exemestane alone stage was significantly associated with marked pathological response. In conclusions, pretreatment Ki67 LI was a predictor of response to neoadjuvant chemo-endocrine therapy. CCND1 expression and ER activity at the post-endocrine therapy alone stage may be useful in determining further treatments.

## Introduction

Estrogen receptor (ER)-positive and human epidermal growth factor receptor 2 (HER2)-negative patients account for 70% of early-stage invasive breast cancer. In current clinical practice, these patients receive either endocrine therapy or chemo-endocrine therapy as adjuvant treatment [1]. The 2017 St. Gallen consensus meeting [2] recommended that adjuvant treatment for ER-positive and HER2-negative breast cancer should be escalated or de-escalated based on the molecular subtype, patient outcome, and side effects of the adjuvant treatment. According to several biomarkers, such as the expression of progesterone receptor (PgR) and the Ki67 labeling index (LI), and several genomic tests (i.e., Oncotype DX and MammaPrint), ER-positive and HER2-negative breast cancer is classified into the luminal A-like (low-risk group), luminal B-like (high-risk group), and intermediate types [2, 3]. The 2017 St. Gallen consensus meeting suggested that luminal A-like breast cancer should be treated with adjuvant endocrine therapy, and luminal B-like breast cancer should be treated with adjuvant chemo-endocrine therapy, respectively [2]. However, the most appropriate treatment for the intermediate type, which accounts for 50% of ER-positive and HER2-negative breast cancer cases [4], remains undetermined. Moreover, several biomarkers have revealed the usefulness to avoid chemotherapy in the low-risk group [4–6]. However, the usefulness of these biomarkers to predict response to chemotherapy in the high-risk group has not been assessed.

Therefore, further investigation of biomarkers to determine the escalation of chemotherapy and/or molecular targeted therapy in ER-positive and HER2-negative breast cancer patients is warranted. In recent clinical trials investigating new molecular targeted therapies, pathological response after neoadjuvant treatment with these agents is used as the endpoint to evaluate the efficacy of the drugs [7, 8]. Numerous studies suggested the pathological response after neoadjuvant treatment as a useful marker for the assessment of response to treatment [9–12]. However; biomarkers predicting pathological response after neoadjuvant chemo-endocrine therapy in ER-positive and HER2-negative breast cancer patients have not been identified.

In the present study, we evaluated the Ki67 LI, expression of PgR and cyclin D1 (CCND1), and level of ER activity in post-menopausal ER-positive and HER2-negative patients at the pretreatment and end of neoadjuvant endocrine therapy alone stages. In addition, the relationship of these factors with pathological response after completing the neoadjuvant chemo-endocrine therapy was assessed.

## Materials and methods

### Patient background and eligibility

**Fig 1:**
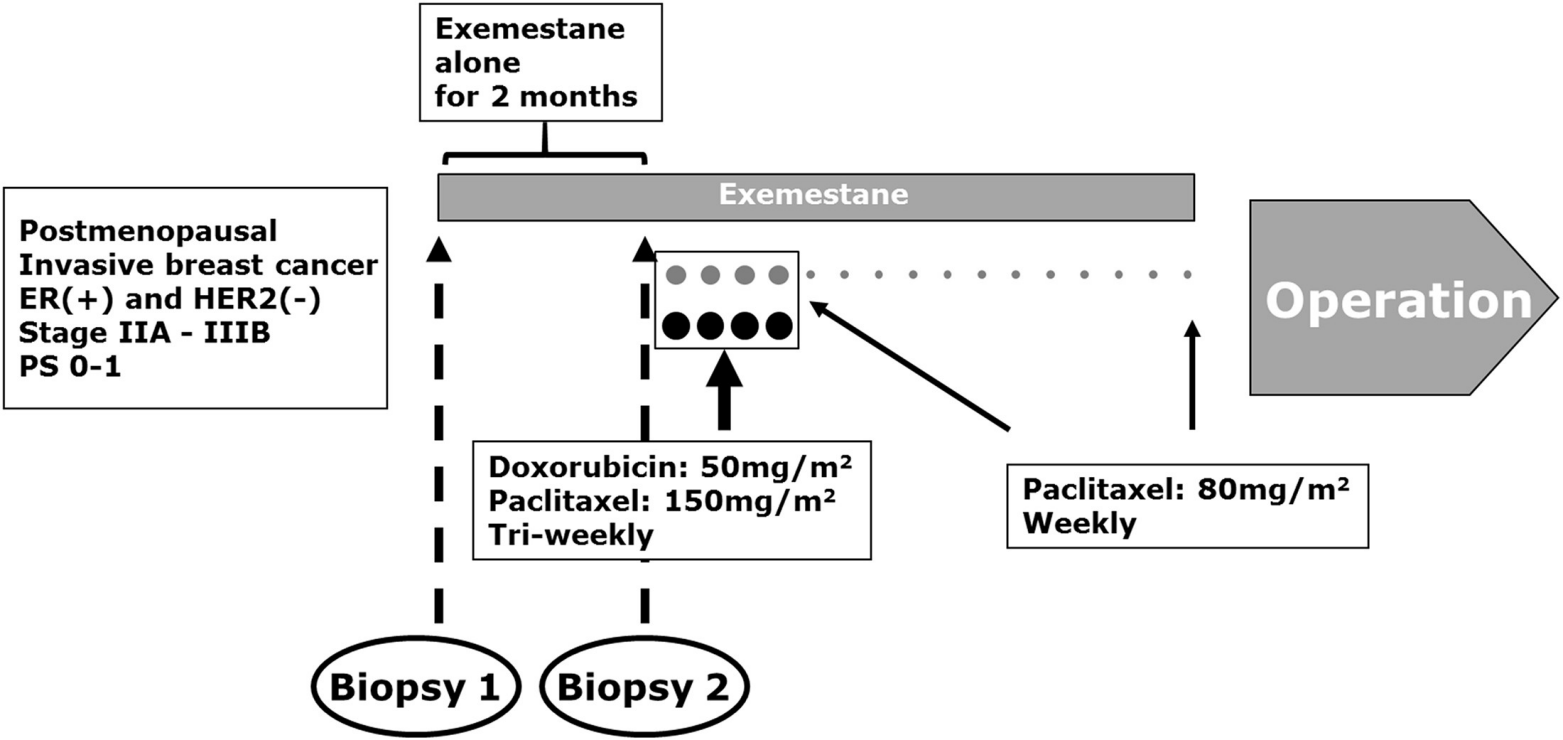
Protocol of the neoadjuvant treatment in the SBCCSG-13 trial. Exemestane alone was administered to all patients for 2 months. Subsequently, the patients received neoadjuvant chemotherapy comprising four cycles of paclitaxel and doxorubicin every 3 weeks followed by 12 cycles of paclitaxel weekly. All patients continued receiving exemestane in this neoadjuvant therapy. The patients enrolled in this study underwent core needle biopsy twice (i.e., pretreatment and after the initial 2 months of therapy with exemestane alone). SBCCSG, Saitama Breast Cancer Study Group.

The present study included 43 post-menopausal female patients with ER-positive and HER-negative invasive breast cancer who received treatment at the Saitama Cancer Center Hospital (Saitama, Japan). Patients with a tumor >2 cm and those who had axillary lymph node metastasis were enrolled. Preoperative treatment with exemestane (25 mg/day) was administered to all patients for 2 months. Subsequently, they received neoadjuvant chemotherapy consisting of four cycles of paclitaxel (150 mg/m^2^) and doxorubicin (50 mg/m^2^) every 3 weeks followed by 12 cycles of weekly paclitaxel (80 mg/m^2^). In addition, all patients received exemestane concomitantly with this neoadjuvant chemotherapy (Fig 1).

This study was conducted in accordance with the principles of the 1964 Declaration of Helsinki and its later amendments or comparable ethical standards. All patients were enrolled in the prospective and multicenter trial of the Saitama Breast Cancer Study Group (SBCCSG) named “Phase II study of exemestane with doxorubicin plus paclitaxel followed by weekly paclitaxel as primary systemic chemotherapy for hormone-sensitive and HER2-negative post-menopausal breast cancer (SBCCSG-13 trial; UMIN: 000001298).” The period of patients’ enrollment was from August 2008 to February 2011. In the SBCCSG-13 trial, core needle biopsy samples were collected pretreatment and post-exemestane alone treatment for the associated translational research (Fig 1). The protocol of the SBCCSG-13 trial and associated translational research were approved by the Institutional Review Board of the Saitama Cancer Center (Reference number: 20080304 and 2015484). All patients enrolled in this study agreed to participate in this study and provided written informed consent.

### Histopathological evaluations

Immunohistochemistry (IHC) and *in-situ* hybridization were performed as previously described [4, 13, 14]. The antibodies were used in IHC staining are as follows: ER (1D5; DAKO, Copenhagen, Denmark), PgR (PgR636; DAKO), and HER2 (HercepTest; DAKO). Amplification of HER2 was achieved using an automated slide-processing system (BenchMark XT; Ventana Medical Systems, Tucson, Arizona, USA) with dual *in-situ* hybridization (DISH; INFORM HER2 Dual ISH DNA Probe Cocktail Assay; Roche, Basel, Switzerland). Specimens with a nuclear staining rate of ≥10% were considered positive for ER. The degree of staining for PgR was assessed using the Allred score. HER2 IHC-evaluation, which classified into four grades (i.e., 0, 1+, 2+, and 3+), was based on the staining intensity of cell membranes. Specimens with a HER2 IHC staining score of 2+ were evaluated for gene amplification using DISH, while those with a HER2 IHC staining score of 3+ or 2+ and positive for HER2 amplification using DISH were identified as HER2-positive breast cancer.

IHC for Ki67 (MIB-1, DAKO) was performed automatically using an automated IHC instrument (BenchMark XT, Ventana Medical Systems, Inc.). The percentage of positivity of Ki67 LI was calculated for approximately 500 tumor cells in the hot-to-warm areas of Ki67-positivity. Ki67 LI and PgR expression were evaluated at the three phases of neoadjuvant treatment: pretreatment (using core needle biopsy samples), post-exemestane alone (using core needle biopsy samples), and post-neoadjuvant therapy (using surgical tissue samples).

The primary CCND1 antibody for IHC (SP4-R) was provided by Roche, Switzerland. Staining was performed automatically using an automated IHC instrument (BenchMark XT, Ventana Medical Systems, Inc.). To determine the expression of CCND1, we evaluated the grades of nuclear staining using the proportion scores of the Allred scoring system. The scores were defined as: 0 (0% staining), 1 (<1%), 2 (1–10%), 3 (10–33%), 4 (33–67%), and 5 (>67%). A score ≥4 denoted high expression of CCND1. The expression of CCND1 at both the pretreatment and post-exemestane alone stages was assessed using core needle biopsy samples.

The pathological response grading to neoadjuvant treatment was performed according to the criteria established by the Japanese Breast Cancer Society. The details of this evaluation have been previously described [14, 15]. In the current study, marked pathological response was defined as grade 3 (no invasive cancer) and grade 2a/b (two-thirds reduction of cancer). Mild response was defined as presence of more than two-thirds of total number of cancer cells (grade 0/1a and grade 1b).

### Evaluation of ER activity

Transfection of an adenovirus vector carrying an estrogen-response element-green fluorescent protein gene (Ad-ERE-GFP) was used to evaluate the level of ER activity. The methodology of this assay has been previously described in detail [16, 17]. Briefly, fresh tumor samples isolated through core needle biopsy were collected. After isolation of tumor cells by digestion with collagenase, 2 x 10^9^ plaque-forming units of Ad-ERE-GFP were transfected into the tumor cells and the cells were incubated at 37°C for 3 days. This process was performed in estrogen (E2)-free medium with phenol-red-free RPMI 1640 supplemented with 10% heat and charcoal-treated fetal calf serum. All experiments were done in triplicate. To examine the infectivity of adenovirus in primary tumor cells, the cells were infected with Ad-CMV-DsRed, and 95% of cells were confirmed to be infected.

The level of ER activity was evaluated in 22 patients at both the pretreatment and post-exemestane alone treatment stages using core needle biopsy samples. GFP-expressing tumor cells were counted using fluorescence microscopy and ER activity was determined by the percentage of GFP-positive tumor cells.

### Statistical analysis

All statistical analyses were conducted using the GraphPad Prism 7.03 (GraphPad Software Inc.) software. The relationships between the Ki67 LI, expression of CCND1 and PgR, and level of ER activity were examined using the Spearman correlation test. The Mann–Whitney test was used for the following comparisons: Ki67 LI and PgR expression between the three phases (i.e., pretreatment, post-exemestane alone, and post-neoadjuvant therapy); CCND1 expression and level of ER activity between the two phases (i.e., pretreatment and post-exemestane alone). The Mann–Whitney test was also used to assess the associations of histological response with Ki67 LI, expression of CCND1 and PgR, and level of ER activity.

## Results

### Patient and tumor characteristics

Patient characteristics are shown in Table 1. Of those enrolled, 18 patients (41.9%) were aged >60 years. Clinical tumor sizes–determined according to the guidelines of the American Joint Committee on Cancer were as follows: T1-2, 35 patients (81.4%) and T3-4, 8 patients (18.6%). The majority (34 patients; 79.1%) were clinical lymph node status-positive. Eleven patients (25.6%) and 34 patients (79.1%) underwent mastectomy and lymph node dissection, respectively.

**Table 1:** Patient and tumor characteristics.

**Age range in year**	
59 and less than	25 (58.1%)
60 and over	18 (41.9%)
**Clinical tumor size**	
Clinical T1-T2	35 (81.4%)
Clinical T3-T4	8 (18.6%)
**Clinical nodal status**	
Negative	9 (20.9%)
Positive	34 (79.1%)
**Type of breast surgery**	
Breast-conserving surgery	32 (74.4%)
Mastectomy	11 (25.6%)
**Axillary surgery**	
Sampling alone	9 (20.9%)
Axillary lymph node dissection	34 (79.1%)
**Pathological response**	
Marked pathological responses	
Grade 3 (pCR)	2 (4.7%)
Grade 2b	1 (2.3%)
Grade 2a	12 (27.9%)
Mild pathological responses	
Grade 1b	18 (41.9%)
Grade 0-1a	10 (23.3%)

Regarding tumor response, 2 patients (4.7%) achieved pathological complete response (pCR) (grade 3). The histological responses of the remaining 41 non-pCR patients were as follows: grade 2b (near-pCR), one patient (2.3%); grade 2a, 12 patients (27.9%); grade 1b, 18 patients (41.9%); and grade 0–1a, 10 patients (23.3%). Marked response (grade 2a-3) and mild response (grade 0–1b) were detected in 34.9% and 65.2% of patients, respectively.

### Analysis according to Ki67 LI and PgR expression

**Fig 2:**
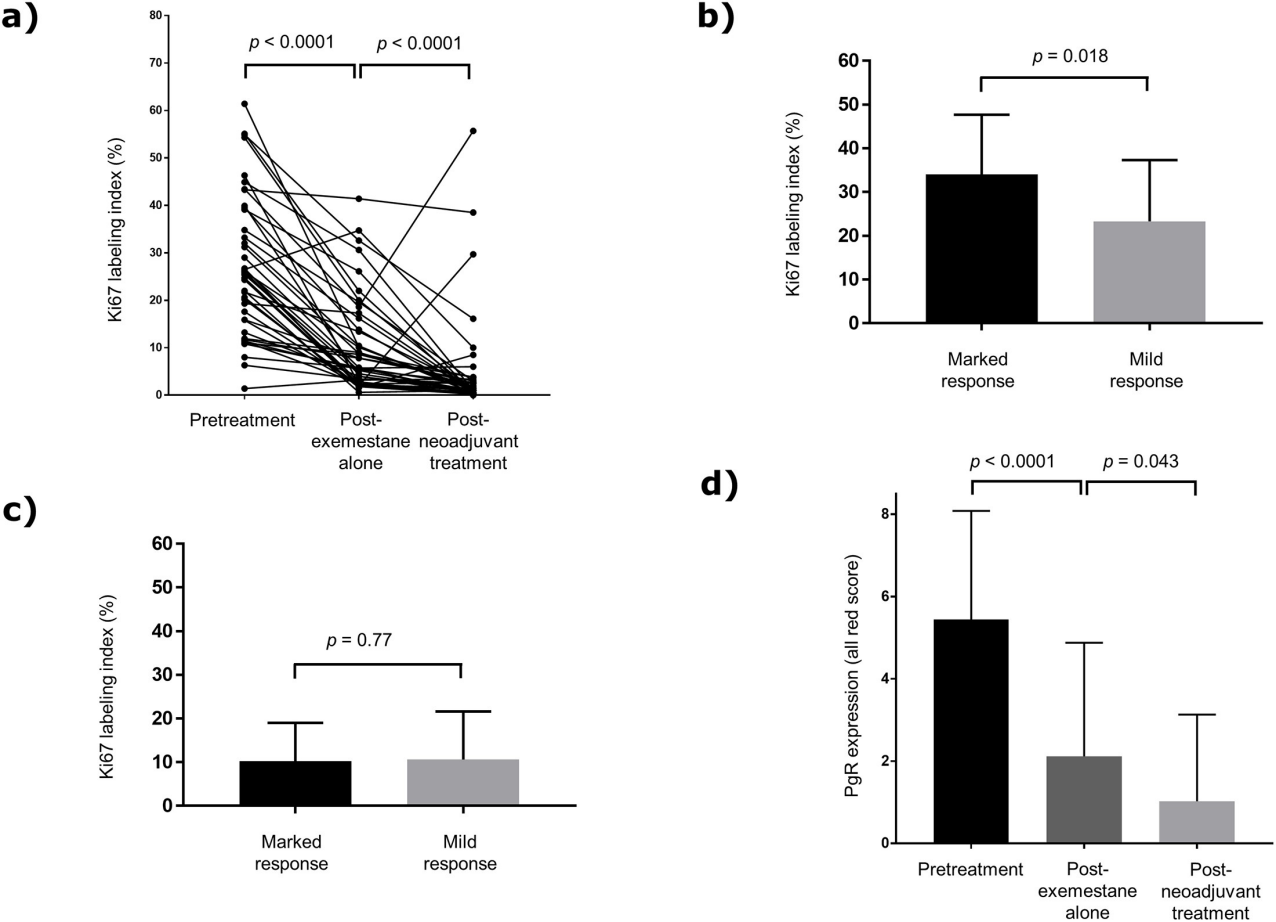
Changes in Ki67 LI and PgR expression in primary and residual tumors after neoadjuvant treatment. (a) Fluctuation of Ki67 LI between the three treatment periods. The Ki67 LI significantly decreased after the neoadjuvant treatment. (b) The comparison of Ki67 LI at pretreatment between patients with marked pathological response and those with mild pathological response. (c) The comparison of Ki67 LI at post-exemestane alone between patients with marked pathological response and those with mild pathological response. Ki67 LI at pretreatment was significantly associated with the pathological response; however, it was not significant at the post-exemestane alone treatment stage. (d) Fluctuation of PgR expression between the three treatment periods. PgR expression significantly decreased after the neoadjuvant treatment. LI, labeling index; PgR; progesterone receptor.

The Ki67 LI was significantly decreased after treatment (Fig 2). Pretreatment high Ki67 LI was a significant predictive factor of marked pathological response (*p* = 0.018). However, it was not significant at the post-exemestane alone treatment stage (Fig 2 and Table 2). The expression of PgR was also significantly decreased after treatment (Fig 2). However, PgR expression was not a significant predictive factor of pathological response at neither of the two assessment stages (Table 2). Moreover, there was no significant correlation between Ki67 LI and PgR expression.

**Table 2:** The relationship of pathological response with Ki67 labeling index, cyclin D1 and PgR expressions, and ER-activity level.

**Pre-treatment**			
**Factors**	**Marked pathological responses group (Median)**	**Mild pathological responses group (Median)**	***p*-value**
**Ki67 labeling index**	32.0	21.0	0.018
**CCND1 expression**	5.0	5.0	0.48
**PgR expression**	6.0	6.5	0.88
**ER-activity**	19.0	20.5	0.47
**Post-exemestane alone**			
**Factors**	**Marked pathological responses group (Median)**	**Mild pathological responses group (Median)**	***p*-value**
**Ki67 labeling index**	10.1	5.8	0.77
**CCND1 expression**	4.0	4.0	0.67
**PgR expression**	0.0	0.0	0.69
**ER-activity**	8.2	25.8	0.028

Abbreviations: CCND1, cyclin D1; PgR, progesterone receptor; ER, estrogen receptor.

### Analysis according to CCND1 expression

The expression of CCND1 was not a significant predictive factor of pathological response at neither the pretreatment nor post-exemestane alone treatment stages (Table 2). In addition, pretreatment, CCND1 expression was not significantly associated with Ki67 LI. However; high CCND1 expression was significantly associated with high Ki67 LI at the post-exemestane alone treatment stage (*p* = 0.011; Table 3). As shown in Table 3, there was no significant correlation between the expression of CCND1 and PgR at neither of the assessment stages.

**Table 3:** The correlation of cyclin D1 expression with Ki67 labeling index and PgR expression.

**Pre-treatment**		
**Factors**	**Correlation with high CCND1 expression**	***p*-value**
**Ki67 labeling index**	-0.02	0.89
**PgR expression**	0.04	0.78
**Post-exemestane alone**		
**Factors**	**Correlation with high CCND1 expression**	***p*-value**
**Ki67 labeling index**	0.38	0.011
**PgR expression**	0.22	0.16

Abbreviations: CCND1, cyclin D1; PgR, progesterone receptor.

**Fig 3:**
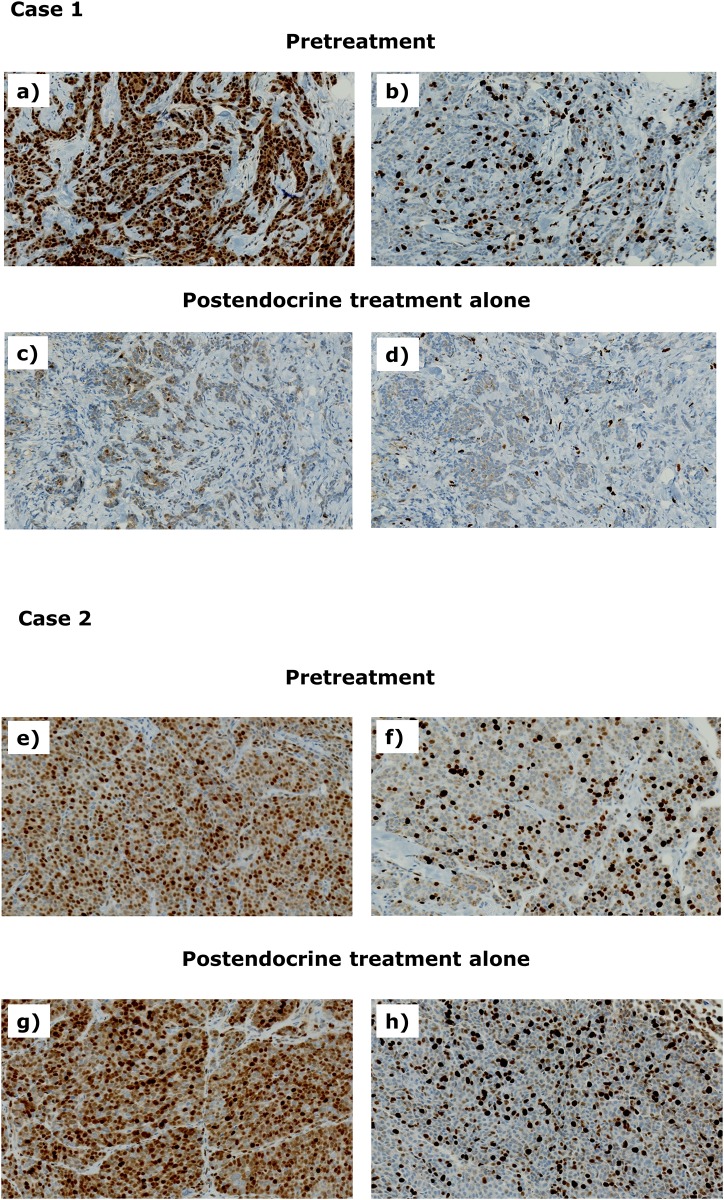
CCND1 expression and Ki67 LI at the pretreatment and post-exemestane alone treatment stages. Case 1: At the pretreatment stage, the patient exhibited high CCND1 expression (a) and high Ki67 LI (b). However, CCND1 expression (c) and Ki67 LI (d) were downregulated at the post-exemestane alone treatment stage. Case 2: At the pretreatment stage, this patient exhibited high CCND1 expression (e) and high Ki67 LI (f). CCND1 expression (g) and Ki67 LI (h) maintained their high levels after the initial 2 months of exemestane alone therapy. LI, labeling index; CCND1, cyclin D1.

Pretreatment, 39 patients (90.7%) and four patients (9.3%) exhibited high and low CCND1 expression, respectively. At the post-exemestane alone treatment stage, these values were 31 (72.1%) and 12 (27.9%), respectively. As shown in Fig 3, among the patients with high CNDD1 expression at the pretreatment stage, the expression was decreased after treatment with exemestane alone in only 10 patients (25.6%).

### Analysis according to ER activity level

**Fig 4:**
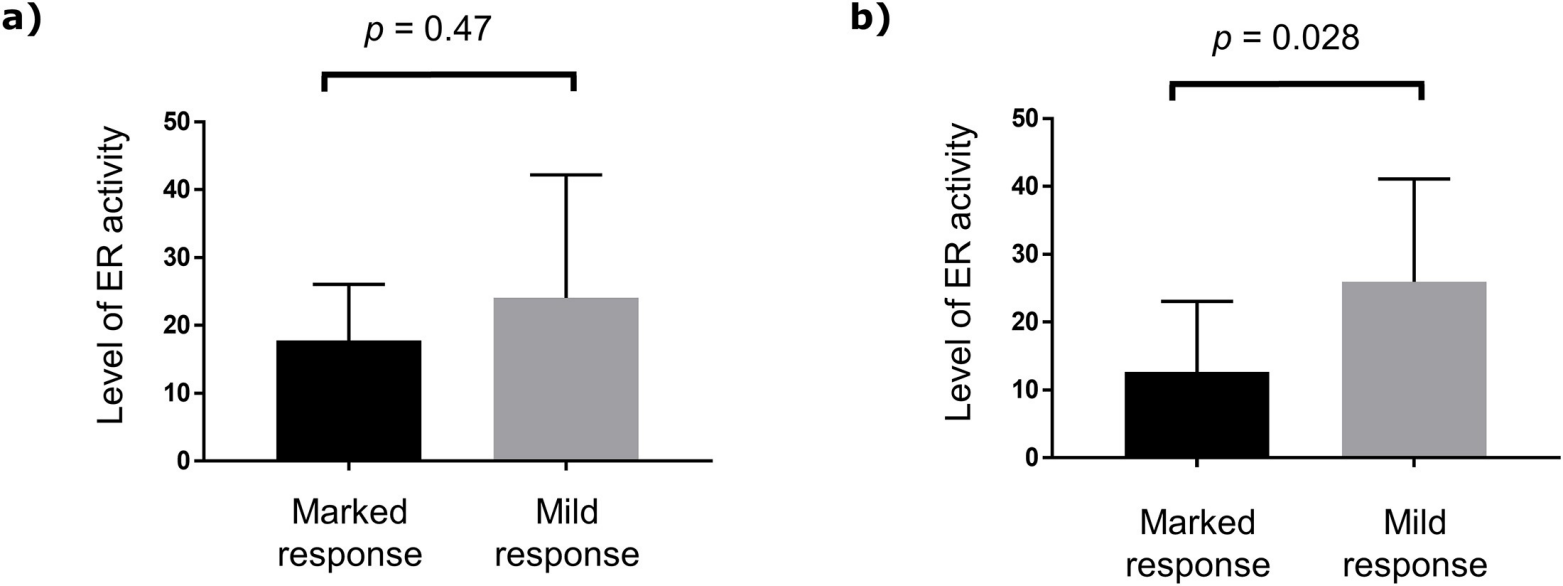
Comparison of the level of ER activity between the patients with marked pathological response and those with mild pathological response. Although the pretreatment level of ER activity was not significantly associated with the pathological response (a), low-level ER activity at the post-exemestane alone treatment stage was associated with marked pathological response after chemo-endocrine therapy (b). ER, estrogen receptor.

The median pretreatment and post-exemestane alone treatment level of ER activity among all patients was 19.3 (index range, 1.0–64.5) and 19.8 (index range, 3.5–59.3), respectively. There was no association of the level of ER activity with Ki67 LI or the expression of CCND1 and PgR at neither of the assessment stages (Table 4). As shown in Table 2 and Fig 4, the pretreatment level of ER activity was not a significant predictive factor of pathological response. However, low-level ER activity at the post-exemestane alone treatment stage was significantly associated with marked pathological response (*p* = 0.028).

**Table 4:** The correlation of ER-activity level with Ki67 labeling index, cyclin D1 expression, and PgR expression.

**Pre-treatment**		
**Factors**	**Correlation with high ER-activity**	***p*-value**
**PgR expression**	-0.03	0.88
**Ki67 labeling index**	-0.14	0.55
**CCND1 expression**	-0.06	0.79
**Post-exemestane alone**		
**Factors**	**Correlation with high CCND1 expression**	***p*-value**
**PgR expression**	0.08	0.72
**Ki67 labeling index**	0.04	0.86
**CCND1 expression**	0.08	0.71

Abbreviations: CCND1, cyclin D1; PgR, progesterone receptor; ER, estrogen receptor.

## Discussion

For many years, factors that could clearly define favorable and poor prognosis have been considered in the design of chemotherapy indication studies addressing early-stage ER-positive and HER2-negative breast cancer. In the 2017 St. Gallen consensus meeting, it was recommended that the multigene assay is the most effective method of evaluation to determine indications for adjuvant chemotherapy–although immunostaining of Ki67 or PgR have also been evaluated as effective methods [2].

Our study showed that pretreatment Ki67 LI was a significant predictive factor for the efficacy of neoadjuvant chemo-endocrine therapy. Ki67 LI is a useful biomarker in evaluating tumor proliferation [18, 19]. Numerous previous trials suggested that pretreatment Ki67 LI may be a useful prognostic factor or predictive factor for the efficacy of neoadjuvant treatment [4, 20–22]. It has been previously reported that Ki67 LI after neoadjuvant endocrine therapy against ER-positive and HER2-negative breast cancer is a useful prognostic factor [23, 24]. Ellis et al. [6] showed that chemotherapy after neoadjuvant endocrine therapy in the low-Ki67 LI group of patients could be avoided. However, the effectiveness of chemotherapy in the high-Ki67 LI group of patients after neoadjuvant endocrine therapy remains unknown. In the present study, only 2 patients (4.7%) achieved pCR. Recent publication by Yu et al [25] with the large cohort found the low pCR rate (7.2%) after neoadjuvant chemo-endocrine therapy in ER-positive and HER2-negaitve breast cancer. Ellis et al [26] suggested the triage to chemotherapy for patients with high Ki67 LI after neoadjuvant endocrine treatment is less effective in the American College of Surgeons Oncology Group Z1031 Trial. In the present study, Ki67 LI following neoadjuvant endocrine therapy was not a predictive factor for the efficacy of neoadjuvant chemo-endocrine therapy. This suggests that new treatment methods–other than chemo-endocrine therapy–may be needed for the high-Ki67 group following neoadjuvant endocrine therapy.

The findings of this study suggest that there is a significant correlation between the expression of CCND1 and Ki67 LI following neoadjuvant endocrine therapy. CCND1 stimulates the cell cycle in breast cancer by binding to cyclin-dependent kinase 4/6 (CDK4/6) [27]. Several recent clinical studies have suggested the efficacy of CDK 4/6 inhibitor in endocrine therapy-resistant metastatic breast cancer [28–30]. Concomitant use of a CDK4/6 inhibitor and endocrine therapy in neoadjuvant treatment is known to markedly decrease Ki67 LI [31]. Johnston et al. [32] showed that, in the PALLET trial involving ER-positive and HER2-negative breast cancer patients, the concomitant use of a CDK4/6 inhibitor and endocrine therapy significantly decreased Ki67 LI compared with endocrine therapy alone. However, there was no difference in clinical response observed between patients treated with concomitant use of a CDK4/6 inhibitor and endocrine therapy and those receiving endocrine monotherapy [32]. Further investigations regarding the efficacy of post-neoadjuvant endocrine therapy with a CDK4/6 inhibitor in the residual high-Ki67LI group of patients with ER-positive and HER2-negative breast cancer are warranted in a clinical trial using CDK 4/6 inhibitor in the neoadjuvant setting.

The level of ER activity is a highly critical index for evaluating the degree of estrogen dependence in breast cancer cells. Gohno et al. [16] developed a GFP-labeling structure by infecting cancer cells with an adenovirus vector carrying cDNA that is coded downstream of the ER ligation point. Moreover, this method has been validated in fresh tumor samples from patients with breast cancer, providing a visual evaluation of the level of ER activity in breast cancer cells [16]. PgR is a protein coded downstream of the ER gene involved in the intracellular genomic pathway controlled by ER [33, 34]. Previously, we reported that the pretreatment expression of PgR is a highly significant prognostic factor in ER-positive and HER2-negative breast cancer [4]. The pretreatment expression of PgR has become a significant prognostic factor in patients receiving neoadjuvant endocrine therapy, although it is not a predictive factor of efficacy [24]. This study showed that despite PgR not being a predictive factor of efficacy in neoadjuvant chemo-endocrine therapy, the efficacy of neoadjuvant chemo-endocrine therapy using an aromatase inhibitor was low in the high-ER activity group after neoadjuvant endocrine therapy. Gohno et al. [16] reported that the level of ER activity is strongly suppressed by the administration of fulvestrant, which is a selective ER degrader (SERD) [35, 36]. Therapy with SERD may be effective in patients with high levels of ER activity after neoadjuvant endocrine therapy with an aromatase inhibitor. Furthermore, Tokuda et al. [17] have suggested that ER activity affects the efficacy of paclitaxel via histone deacetylase 6 (HDAC6). Therefore, further functional research is warranted to investigate the efficacy of concomitant therapy using an HDAC inhibitor with chemo-endocrine therapy in the high ER-activity group, after neoadjuvant endocrine therapy with an aromatase inhibitor.

In conclusions, the expression of CCND1 and ER activity following neoadjuvant endocrine therapy may be associated with the efficacy of chemo-endocrine therapy in post-menopausal patients with ER-positive and HER2-negative breast cancer. Certain patients in this group cannot be clearly determined as Luminal A-like or Luminal B-like types. In such cases, it may be necessary to consider the escalation of subsequent treatment through combination with chemotherapy or a new molecular targeted drug. This refers to the degree of cell cycle activation and level of ER-activity after neoadjuvant endocrine therapy with an aromatase inhibitor.
